# Intrathyroid injection of dexamethasone inhibits Th2 cells in Graves’ disease

**DOI:** 10.20945/2359-3997000000244

**Published:** 2020-06-05

**Authors:** Ke He, Peng Jiang, Bing-li Liu, Xiao-mei Liu, Xiao-ming Mao, Yun Hu

**Affiliations:** 1 Department of Endocrinology Wuxi Hospital of Traditional Chinese Medicine Nanjing University of Chinese Medicine Wuxi China Department of Endocrinology, Wuxi Hospital of Traditional Chinese Medicine, Wuxi Hospital Affiliated to Nanjing University of Chinese Medicine, Wuxi, China; 2 Department of Thyroid and Breast Surgery Nanjing First Hospital Nanjing Medical University Nanjing China Department of Thyroid and Breast Surgery, Nanjing First Hospital, Nanjing Medical University, Nanjing, China; 3 Department of Endocrinology Nanjing First Hospital Nanjing Medical University Nanjing China Department of Endocrinology, Nanjing First Hospital, Nanjing Medical University, Nanjing, China

**Keywords:** Dexamethasone, Graves’ disease, T help cells, Chemokine

## Abstract

**Objective:**

Intrathyroid injection of dexamethasone (IID) was used for decrease the relapse rate of hyperthyroidism in the treatment of Graves’ disease (GD), but the mechanism is still unclear. We aimed to explore the effect of IID on T help (Th)1/Th2 cells and their chemokine in patients with GD.

**Subjects and methods:**

A total of 42 patients with GD who were euthyroidism by methimazole were randomly divided into IID group (n = 20) and control group (n = 22). Thyroid function and associated antibody, Th1/Th2 cells proportion, serum CXCL10 and CCL2 levels, and CXCR3/CCR2 mRNA expression in peripheral blood mononuclear cells before and after 3-month IID treatment were tested by chemiluminescence assay, Flow cytometry, ELISA, and real-time PCR, respectively. Thyroid follicular cells were stimulated by IFN-γ and TNF-α and treated with dexamethasone in vitro. CXCL10 and CCL2 levels in supernatant were determined.

**Results:**

After 3-month therapy, the proportion of Th2 cells and serum CCL2 levels, as well as TPOAb, TRAb levels and thyroid volume decreased in IID group (p < 0.05). However, the proportion of Th1 and CXCL10 levels had no change in IID group and control (p > 0.05). The CXCR3/CCR2 ratio had no change in both groups (p > 0.05).

**Conclusion:**

IID therapy could inhibit peripheral Th2 cells via decreasing CCL2 level in peripheral blood, and this result partly explain the effects of IID therapy on prevention of relapse of GD. Arch Endocrinol Metab. 2020;64(3):243-50

## INTRODUCTION

Graves’ disease (GD) is a thyroid-specific autoimmune disease that thyroid-stimulating antibody (TSAb) stimulates thyroid-stimulating hormone receptor (TSHR) and leads to overproduction of thyroid hormones. Lymphocytic infiltration is one of the histopathological hallmarks of GD. Previous studies have suggested that the pattern of CD4^+^ T cell in thyroid tissue changes throughout the course of GD and is partially responsible for the pathogenesis of the disease. Although GD was thought to be a typically Th2(T help 2) cells-response-mediated disorder ( 1 , 2 ), some studies have proven that the Th1 (T help 1) cells were dominance in GD patients in the initial phase of GD ( 3 , 4 ), which were strongly associated with cell mediated immune responses, IFN-γ and TNF-α are secreted by Th1 cells that enhance the DNA binding activity of NF-κB and activate the inflammation reaction in thyroid follicular cells, which were deemed to play pivotal roles in the pathogenesis of GD ( 5 ). Moreover, TSAb is predominantly immuno-globulin G (IgG) 1 subclass, which is mediated by Th1 cytokines in humans ( 6 ). However, a progressive transition from Th1 to Th2 dominance appeared, the CXCR3 (Th1-associated chemokine receptor)/CCR4 (Th2-associated chemokine receptor) ratio in peripheral blood mononuclear cells (PBMCs) and IL-12/IL-15 (secreted by Th1/Th2 cell respectively) were decreased after treatment with methimazole (MMI) in GD patients ( 3 , 7 ). Furthermore, the relapse of GD after MMI treatment reported to be more frequent after an attack of allergic rhinitis ( 8 ), and TRAb increased in patients with bronchial asthma or pollen allergy ( 9 ). All of these diseases elevate serum IgE and interleukin (IL)-13 levels, which consistent with a Th2 response ( 9 , 10 ). Therefore, we supposed that Th2 cells may contribute to the high relapse ratio in the GD patients after MMI withdraw.

Several studies showed a significant increase in Chemokine (C-X-C motif) ligand 10 (CXCL10, also named IP-10) strongly associated with Th1-mediated immune responses in thyroid tissue specimens and serum obtained from recent-onset GD patients ( 9 , 10 ). The CXCL10 can be induced by IFN-γ and TNF-α *in vitro* ( 11 , 12 ). Chemokine (C-C motif) ligand 2 (CCL2, also named MCP-1) is a chemokine that attracts Th2 cells and can be produced by thyroid follicular cells ( 13 , 14 ). Increased level of CCL2 has been observed both in peripheral blood of patients with autoimmune thyroid disease (AITD) ( 15 ) and supernatant of thyroid follicular cells from GD patients after stimulated by IFN-γ and TNF-α *in vitro* (12. Yamazaki and cols. found that CCL2 was induced by high concentration of iodide in thyroid follicular cells (16).

Dexamethasone (DEX) is one of the most used glucocorticoids to treat Graves’ ophthalmopathy and hyperthyroidism crisis. In our previous study, an intrathyroid injection of DEX (IID) could effectively reduce the relapse rate in GD patients after withdrawing MMI treatment ( 17 ). The serum thyrotropin receptor antibody (TRAb) levels, the TRAb positive rate, and thyroid volume also decreased after the treatment ( 17 ). We treated peripheral blood mononuclear cells (PBMCs) isolated with DEX from GD patients *in vitro* and found that DEX could decrease the proportion of Th2 cells ( 18 ). However, the effects and mechanisms of IID on Th1/Th2 cells in GD patients are still unclear. The present trial was designed to determine the effect of IID on Th1/Th2 cells in patients with GD and investigated the modulation of CXCL10, CCL2 by DEX.

## SUBJECTS AND METHODS

### Patients

This study was a randomized control trial. The institutional review board at Nanjing First Hospital Affiliated to Nanjing Medical University approved the study protocol (available with the full text of this article at NEJM.org). All patients provided written informed consent. The methods were carried out in accordance with the Declaration of Helsinki guidelines, including any relevant details. The clinical trial registration number of IID therapy was NCT01534169 (https://register.clinicaltrials.gov.)

A total of 50 eligible patients (20 to 54 years of age) with GD (19), including clinically and biochemically verified hyperthyroidism and positive thyrotrophin receptor antibody (TRAb), were recruited in this study from the Endocrine Clinic of Nanjing First Hospital Affiliated to Nanjing Medical University between June 2011 and March 2012. The clinical evaluation included the patient’s history, physical examination, and thyroid ultrasound. The laboratory diagnosis included serum levels of free T4_,_ (FT4), sensitive TSH (s-TSH) and TRAb. All of the patients received MMI treatment with a titration regimen for 0-18 months until a condition of euthyroidism was achieved (euthyroidism was defined as the elimination of most symptoms of hyperthyroidism and serum levels of TSH and FT4 in the normal range), and a continuing dose of MMI was given to maintain euthyroidism as a background therapy. Major exclusion criteria were patients with other coexistent endocrine or organ-specific autoimmune disease, taking medications or had a medical history (such as corticosteroids) that could affect the immune system, pregnancy, allergy to ATD, alanine aminotransferase (ALT) or aspartate aminotransferase (AST) levels more than two times the upper normal range, noncompliance because of psychiatric or other serious diseases, or unwillingness to participate in the study.

### Detection of thyroid hormones and anti-thyroid antibodies

Fasting peripheral blood was obtained from patients with GD before and after the treatment, Serum TSH (reference range, 0.55-4.78 mIU/L) and FT4 (reference range, 11.5-22.7 pmol/L) levels were measured by chemiluminescence assay (Centaur XP automated chemiluminescence immunoassay analyzer, Siemens, Germany). TRAb (reference range, < 1.75 IU/L), thyroid peroxidase antibody (TPOAb) (reference range, < 34 IU/mL) and thyroglobulin antibody (TGAb) (reference range, <115 IU/mL) levels were assessed by electrochemical luminescence assay (Roche E170 immune analyzer, Roche, Switzerland). Thyroid volume was obtained by computing the volumes of both lobes by color Doppler ultrasound. Lobe (mL) = length (mm) × width (mm) × depth (mm) × 0.479. Nodules and/or cystic areas were included in the thyroid volume.

### Intrathyroid injection of DEX (IID) therapy

Patients were enrolled in the trial between February, 2012 and November 2015 at Endocrine Clinic of Nanjing First Hospital. In the IID treatment group, patients received intrathyroid injection of DEX (Tianjin Pharmaceutical Jiaozuo Co, China) at doses of 5 mg (1.0 mL) in each lobe, twice a week during the first month of the treatment. The treatment strategy was changed to once a week in the second month and twice a month in the third month, the dosage of DEX was the same as in the first month. This treatment procedure was conducted under ultrasound guidance, the details of this treatment process were the same as our previous study ( 17 ). In the control group, patients continued MMI treatment and the dose was unchanged. Blood from all of the patients was collected before and after the 3-month therapy, Th1/Th2 cells were tested immediately, while serum was kept frozen at –80 °C until the thyroid hormones, anti-thyroid antibodies, CXCL10 and CCL2 were tested.

### RNA isolation and real-time quantitative PCR of chemokine receptors in PMBCs

Total RNA was isolated from PMBCs using TRIzol reagent (Ambion; Life Technologies). The CXCR3 and CCR2 mRNA expressions were quantified by real-time PCR using ABI Prism 7500 Sequence Detector (Applied Biosystems; Life Technologies). RT and PCR were performed (SYBR^®^ PrimeScript^®^ RT-PCR Kit; Takara Bio, Inc., Otsu, Shiga, Japan) using primer for CXCR3 designed as follows: 5’- TGGCCGAGAAAGCAGGG-3’ and5’- AGGCGCAAGAGCAGCATC-3’. The primer set for CCR2 consisted of 5’- AGTTCAGAAGGTATCTCTCGGTG-3’ and 5’- GGCGTGTTTGTTGAAGTCACT-3’. The primer set for β-actin consisted of 5’-ATCTGCTGGAAGGTGGACAGCGA-3’ and 5’-CCCAGCACAATGAAGATCAAGATCAT-3’. The total reaction volume was 20 μL and the PCR was programmed as an initial incubation for 30s at 95 °C followed by 40 thermal cycles of 5s at 95 °C and 34s at 60 °C. The relative quantity of CXCR3 and CCR2 mRNA before and after IDD therapy was calculated by using the equation 2^-^DD^Ct^. All reactions were confirmed by at least one additional independent run.

### Flow cytometry analysis

Heparinized blood was collected from patients. Th1/Th2 cells were stimulated and stained as the protocol of Th1/Th2 cells test kit (BD Biosciences), using anti-CD3-PerCP-Cy5-5, anti-CD8-PE-Cy7 to identify Th cells. Th1 and Th2 cells were stained with anti-IFN-γ-FITC anti-IL-4-PE, respectively.

### Thyroid follicular cells

Surgical thyroid tissue was obtained from 4 patients (2 females and 2 males, age range 33-56 years) who underwent surgery for multinodular goiters in Nanjing First Hospital from January, 2016 to June, 2017. Certificates of consent were obtained. Patients did not receive any specific treatment for thyroid disease; thyroid hormones and thyroid autoantibody measurements were in the normal range.

Thyroid follicular cells were prepared as previously described ( 20 ). The tissues were minced to fragments as small as possible and digested by 250 u/mL collagenase II and 0.25% trypsin (both from Gibco, USA) in D-Hank’s for 90 min at 37 °C. Digested tissues were mechanically dispersed until a homogeneous suspension was obtained. After washing with D-Hank’s, the cell suspension was cultured in DMEM medium containing 10% fetal bovine serum (Gibco, USA), 2 mM glutamine, and 50 μg/mL penicillin/streptomycin at 37 °C and 5% carbon dioxide. Cells were used within the forth to sixth passage.

### Cytokines stimulates and DEX treatment *in vitro*

Thyroid follicular cells were seeded at a density of 2×10^4^ cells per milliliter in 96-well plates in 200 μL volume media. After 48 hours, the medium was removed; and cells were washed with phosphate-buffered saline (PBS) and incubated in phenol red and serum-free medium. Cells were incubated (24 hours) with or without a combination of 1,000 U/mL IFN-γ and 10 ng/mL TNF-α (both from Sigma-Aldrich) as reported previously ( 21 ) in the presence or absence of DEX. The supernatant was harvested and kept frozen at –20 °C until CXCL10 and CCL2 tested.

### Methylthiazol tetrazolium (MTT) assay

Methylthiazol tetrazolium (MTT) assay was used to determine the terminal concentrations of DEX. Thyroid follicular cells were seeded at a density of 2×10^4^ cells per milliliter in 96-well plates in 200 μL volume media. DEX was added in different doses (0, 10^-4^, 10^-5^, 10^-6^mol/L). The thyroid follicular cells were cultured for 12 h, 24 h, and 48 h, and then 10 μL of MTT solution (5 mg/mL in PBS) was added into each well. Then cells were incubated at 37 ºC for 4 hours allowing the MTT to be metabolized. The supernatant was removed and 100 μL of DMSO was added into each well to dissolve formazan crystals. The absorbance of the solutions with dye was measured at 492 nm on a multi-well spectrophotometer (BioTek, Winooski, VT, USA).

### Enzyme-linked Immunol Sorbent Assay (ELISA)

CXCL10 and CCL2 levels in serum and culture supernatants were assayed by a quantitative sandwich immunoassay using an ELISA kit (R&D Systems, Inc., Minneapolis, MN). The intra- and inter-assay coefficients of variation were 3.0% and 6.7% for CXCL10, and 4.5% and 6.0% for CCL2, respectively.

### Statistical analysis

All the experiments were run in triplicate. Statistical analysis was performed using SPSS 16.0 Software (SPSS, Inc., Chicago, IL, USA). Data are expressed as mean ± S.E.M. The Kolmogorov–Smirnov test was used to test for normal distribution of the data. Proportions were compared by the chi-square test. The differences between two groups were analyzed using the two-tailed Student’s t-test for normally distributed variables, or either the Mann-Whitney U or Wilcoxon test for nonparametric variables. A P value < 0.05 was considered significant.

## RESULTS

### The clinical characteristics of GD patients

**Figure 1:**
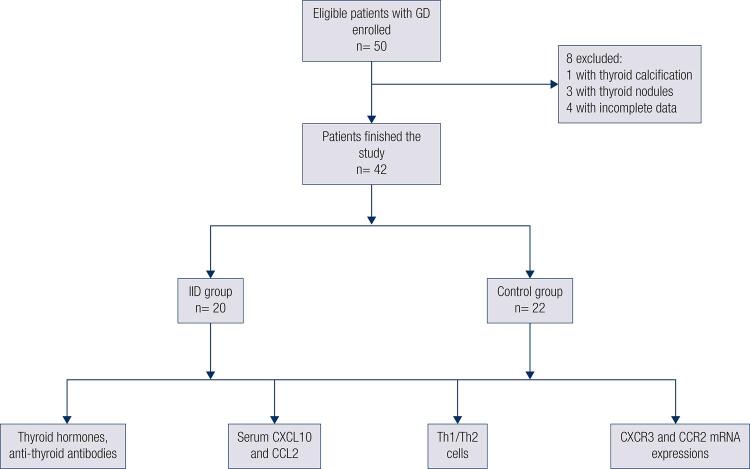
Patients with GD enrolled in the study.

A total of 42 patients finally finished the study. One of the patients with thyroid calcification and 3 patients with thyroid nodules found by ultrasound before IID therapy were lost. The other 4 patients were excluded because of incomplete data ( Figure 1 ). The clinical characteristics of patients before and after the therapy are shown in Table 1 , and there was no significant difference between control and IID groups at the beginning of the therapy. After 3-month therapy, TSH, FT4, TGAb, TPOAb, TRAb and thyroid volume had no change in control while the TPOAb, TRAb and thyroid volume decreased in IID group (p < 0.05).

**Table 1:** The clinical characteristics of patients before and after the therapy

Number	Control group		IID group	
	
	Baseline	After therapy	Baseline	After therapy
	
	22		20	
Age (year)	36.00 ± 3.06		32.80 ± 3.99	
Gender (male)	13.63%		10.00%	
Duration of GD (month)	12.92 ± 0.76		10.80 ± 1.48	
TSH (mIU/L)	1.38 ± 0.21	1.87 ± 0.27	1.44 ± 0.25	2.40 ± 0.35
FT4 (pmol/L)	17.16 ± 0.67	15.99 ± 0.94	15.84 ± 0.74	13.79 ± 0.75
TGAb (IU/mL)	704.22 ± 329.08	618.79.26 ± 325.24	613.03 ± 302.23	465.43 ± 265.72
TPOAb (IU/mL)	228.93 ± 66.53	213.26.21 ± 66.07	220.18 ± 57.45	150.40 ± 51.54*
TRAb (IU/L)	7.44 ± 3.13	6.82 ± 2.93	9.11 ± 2.59	4.65 ± 1.62*
Thyroid volume (mL)	20.60 ± 6.06	20.82 ± 5.63	22.29 ± 3.29	18.56 ± 2.84*

* p < 0.05 vs. baseline using the two-tailed Student’s t-test. IID: intrathyroid injection of dexamethasone; GD: Graves’ disease; TSH: thyroid-stimulating hormone; FT4: free thyroxin T4; TGAb: thyrotrophin receptor antibody; TPOAb: thyroid peroxidase antibody; TGAb: thyroglobulin antibody.

### Proportion of circulating Th1/Th2 cells in GD patients with IID therapy

**Figure 2:**
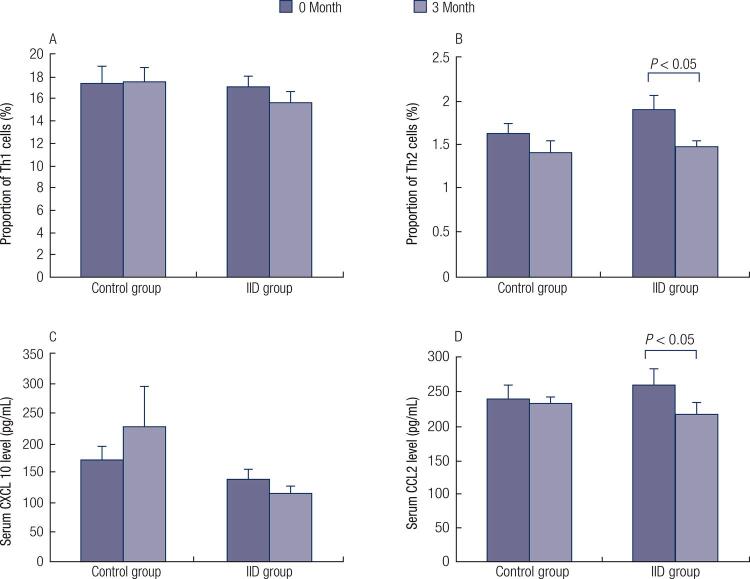
A. Proportion of Th1 cells in circulating CD3+ T cells before and after 3-month therapy tested with flow cytometry. B. Proportion of Th2 cells in circulating CD3+ T cells before and after 3-month therapy. C. Serum CXCL10 level in GD patients in control and 3-month group before and after IID therapy tested by ELISA. D. Serum CCL2 level in GD patients in control and IID group before and after IID therapy.

Proportions of Th1/Th2 cells in circulating CD3^+^ T cells were tested by flow cytometry. There were no differences in Th1 and Th2 proportion between control group and IID group at the beginning of therapy. After 3-month of therapy, the proportions of Th1 cells in IID group and control group were similar. However, Th2 cells decreased in IID group, while no significant difference was found before and after 3-month of therapy in the control group, see in Figure 2A, B .

The CXCR3/CCR2 in PMBCs were also performed by PCR to show the shift of Th1/Th2 cells balance in peripheral blood. No significant difference was found between IID and control group before or after the treatment. The CXCR3/CCR2 ratio in control group and IID was 1.59 ± 0.37 and 1.17 ± 0.29, respectively (p > 0.05).

### Modulation of serum CXCL10 and CCL2 levels in GD patients with IID therapy

Serum CXCL10 and CCL2 levels from GD patients before and after the therapy were tested by Elisa. Before the therapy, serum CXCL10 and CCL2 levels were similar in control and IID groups. After 3-month therapy, the CXCL10 level had no significant change in the two groups (p > 0.05), while CCL2 level decreased significantly in IID group (p < 0.05), see in Figure 2 C, D .

### Effects of DEX on CXCL10 and CCL2 release of thyroid follicular cells *in vitro*

We then evaluated the effects of DEX on CXCL10 and CCL2 release of thyroid follicular cells stimulated by IFN-γ and TNF-α. DEX was co-cultured with thyroid follicular cells for 24 h. The terminal concentrations of DEX were all 10^-5^ mol/L. The dose was determined by the results of the MTT assay.

**Figure 3:**
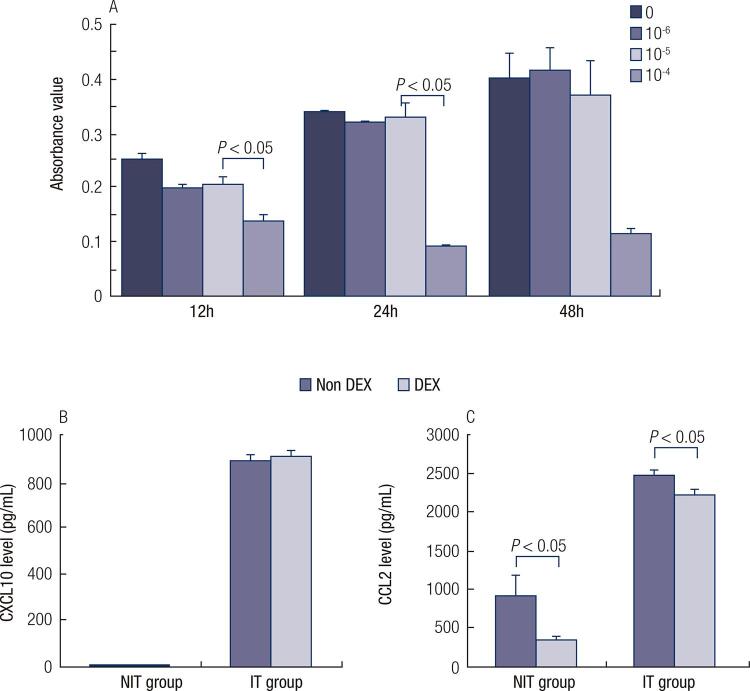
A. Different doses of DEX (0, 10-4, 10-5, 10-6 mol/L) were treated on thyroid follicular cells after stimulated with IFN-γ and TNF-α. Then MTT assay was used to estimate cell growth viability at 12 h, 24 h and 48 h. B. CXCL10 concentration of thyroid follicular cells supernatant in NIT (Non IFN-γ and TNF-α) group and IT (IFN-γ and TNF-α stimulated) group tested by ELISA assay, with or without DEX treatment for 24h. C. CCL2 concentration of thyroid follicular cells supernatant in NIT group and IT group tested by ELISA assay, with or without DEX treatment for 24h.

The MTT assay showed that higher concentration of DEX caused cell death, see in Fig 3A. Both of CXCL10 and CCL2 levels in the supernatant of thyroid follicular cells increased significantly after IFN-γ and TNF-α stimulation (p < 0.001). There was no significant difference of CXCL10 level between the groups with or without DEX treatment. In contrast, CCL2 decreased significantly after DEX treatment (p < 0.05), see in Figure 3B, C .

## DISCUSSION

This study confirmed the findings of previous studies ( 12 , 22 ) that IFN-γ plus TNF-α stimulated CXCL10 and CCL2 secretion of thyroid follicular cells *in vitro* . And this study showed, for the first time, that DEX inhibited IFN-γ plus TNF-α induced CCL2 secretion, but had no effect on CXCL10, this result was obtained both in GD patients after IID therapy and in primary thyroid follicular cells *in vitro* . Th2 cells decrease in peripheral blood by DEX after IID therapy. Meanwhile, the TPOAb, TRAb and thyroid volume were also decreased, which was found in previous study ( 17 ).

CCL2 produced by infiltrated inflammatory cells and/or thyroid follicular cells may play an important role in thyroid immune/inflammatory responses. It attracts monocytes, memory T lymphocytes, and natural killer cells *in vitro* ( 23 ) and may regulate T-cell differentiation ( 24 ). Antonelli and cols. showed that IFN-γ and TNF-α, alone or in combination, stimulated the secretion of the CCL2 in primary thyroid follicular cells from patients with GD and normal person ( 12 ). The results indicate that CCL2 can be produced by thyroid follicular cells themselves under the influence of cytokines such as IFN-γ or TNF-α, which are released by activated Th1 lymphocytes and confirm the hypothesis that autoimmune disorders in thyroid of GD patients evolve from an initial Th1 phase to a later Th2 prevalent immune response.

The effect of DEX on CCL2 has been found in other diseases and tissues by several studies ( 25 - 27 ). These studies showed that DEX inhibited both increases of CCL2 and CXCL10 which could induced by hydrogen peroxide in neurons ( 25 ) or by IFN-γ in endothelial cells ( 26 ). However, DEX has no significant effect on CXCL10 in thyroid cells in this study. The difference may be contributed to the different tissues. The effect of DEX on CCL2 is mediated by the glucocorticoid receptor (GR) and involves an apparently novel mechanism in which the GR binds directly to CCL2 mRNA and facilitates its degradation ( 28 , 29 ). As a result, the recruitment of Th2 cells by CCL2 in thyroid can be inhibited by IID therapy in GD patients, and Th2-mediated immune responses are decreased. Th2 cells proportion of CD3^+^ cells in peripheral blood was lower after 3-month IID therapy, but the CCR2 (CCL2 receptor in Th2 cells) did not decrease significantly. The Th2 cells, overall, were inhibited slightly in peripheral blood after IID therapy and may be more significant in thyroid, because DEX was injected into thyroid locally.

As we expected, the level of TPOAb, TRAb and thyroid volume decreased after IID treatment in patients with GD, which was in accordance with our previous study ( 17 ). The reduction of TPOAb and TRAb might be due to the immunoregulatory effects of DEX, like as improvement of the Tregs function ( 18 ), and inhibition of the Th2-mediated immune responses. The reduction of thyroid volume might be owing to the apoptosis-promoting effect of DEX on thyrocyte directly which were observed in thymocytes ( 30 ). Moreover, the Th2 cytokine profile, which is inhibited by DEX, is associated with upregulation of antiapoptotic molecules, rendering the thyrocytes but not the infiltrating lymphocytes immune from apoptosis in GD ( 31 ).

There are some reasons why we use IID scheme to treat GD patients. The first, GD is an organ-specific autoimmune disease, the main pathological reaction occur in thyroid. The IID can keep a relative higher concentration of DEX in thyroid gland. The second, low dose of DEX was needed to perform this treatment, which can reduce the side effects of DEX therapy. In the present study, no overt systemic adverse reactions induced by DEX were found.

Our study has several limitations. As well as all of the other studies about the shift of Th1/Th2 cell balance and changes in chemokine secreted by thyroid follicular cells, this study was conducted in peripheral blood but not thyroid. The changes of Th1/Th2 cells balance and the CCL2/CXCL10 levels in thyroid after IID therapy can not be observed directly. Moreover, the sample size was small, which frustrated the analysis of Th1/Th2 cells balance in different gender and age.

In conclusion, our study showed that IID therapy could inhibit peripheral Th2 cells via decreasing CCL2 levels in peripheral blood, and this result partly explain the effects of IID therapy on prevention of relapse of GD.
